# Statistical Reporting Errors and Collaboration on Statistical Analyses in Psychological Science

**DOI:** 10.1371/journal.pone.0114876

**Published:** 2014-12-10

**Authors:** Coosje L. S. Veldkamp, Michèle B. Nuijten, Linda Dominguez-Alvarez, Marcel A. L. M. van Assen, Jelte M. Wicherts

**Affiliations:** 1 Department of Methodology and Statistics, Tilburg School of Social and Behavioral Sciences, Tilburg University, Tilburg, The Netherlands; 2 Department of Organization Studies, Tilburg School of Social and Behavioral Sciences, Tilburg University, Tilburg, The Netherlands; University of Geneva, Switzerland

## Abstract

Statistical analysis is error prone. A best practice for researchers using statistics would therefore be to share data among co-authors, allowing double-checking of executed tasks just as co-pilots do in aviation. To document the extent to which this ‘co-piloting’ currently occurs in psychology, we surveyed the authors of 697 articles published in six top psychology journals and asked them whether they had collaborated on four aspects of analyzing data and reporting results, and whether the described data had been shared between the authors. We acquired responses for 49.6% of the articles and found that co-piloting on statistical analysis and reporting results is quite uncommon among psychologists, while data sharing among co-authors seems reasonably but not completely standard. We then used an automated procedure to study the prevalence of statistical reporting errors in the articles in our sample and examined the relationship between reporting errors and co-piloting. Overall, 63% of the articles contained at least one *p*-value that was inconsistent with the reported test statistic and the accompanying degrees of freedom, and 20% of the articles contained at least one *p*-value that was inconsistent to such a degree that it may have affected decisions about statistical significance. Overall, the probability that a given *p*-value was inconsistent was over 10%. Co-piloting was not found to be associated with reporting errors.

## Introduction

Most conclusions in psychological research (and related fields) are based on the results of Null Hypothesis Significance Testing (NHST) [1], [2], [3], [4], [5], [6]. Although the use and interpretation of this method have been criticized (e.g. [7], [8], [9]), it continues to be the main method of statistical inference in psychological research [10], [11]. Not only for the readers of the psychological literature to be able to interpret and assess the validity of research results, but also for the credibility of the field, it is thus crucial that NHST results are correctly reported. Recent results however suggest that reported results from *t*, *F*, and χ^2^ tests in the scientific literature are characterized by a great deal of errors [11], [12], [13], [14], [15]. An example of such an error can be found in the following results (which, apart from the variable names, appeared in a published article): “All two-way interactions were significant: A×B, *F*(1, 20) = 9.5, *p*<.006; A×C, *F*(1, 20) = 0.54, *p*<.03; and C×B, *F*(1, 20) = 6.8, *p*<.02″. Even without recalculation, the experienced user of NHST may notice that the second of these *p*-values is inconsistent with the reported F-statistic and the accompanying degrees of freedom. The *p*-value that corresponds to this F-statistic and these degrees of freedom equals.47. Bakker and Wicherts [11] found that 50% of the articles reporting the results of NHST tests in the psychological literature contained at least one such inconsistent *p*-value, and that 18% of the statistical results was incorrectly reported. Similar yet slightly lower error rates have been found in the medical literature [12], [13] and in recent replications [15], [16], [17]. Bakker and Wicherts [11] discuss different reasons why these inconsistent *p*-values may appear. For example, the output for a three-way Analysis of Covariance (ANCOVA) in the current version of the popular package SPSS contains no less than 79 numbers, many of which are redundant and therefore easily incorrectly retrieved. When several analyses are conducted, results are readily mixed up and typographic errors quickly occur. Other reasons for statistical errors may be misunderstanding of data analysis in general [18] or misunderstanding of NHST [6] in particular.

In many areas where human errors are common and potentially consequential, systems have been implemented to help reduce the likelihood of these errors [19]. An example of such a system is co-piloting in aviation: double-checking the pilot’s every move significantly reduces the risk of human errors leading to airplane crashes [20], [21]. Another example is pair-programming in Agile Software Engineering, which is found to help reduce errors in programming code [22]. Wicherts [23] suggested that scientists should learn from aviation and other fields that deal with human error, and proposed a method to reduce errors in the reporting of statistical results: the co-pilot model of statistical analysis. This model involves a simple code of conduct prescribing that statistical analyses are always conducted independently by at least two persons (typically co-authors). This would stipulate double-checks of the analyses and the reported results, open discussions on analytic decisions, and improved data documentation that facilitates later replication of the analytical results by (independent) peers.

Contrary to common practice in medical sciences where statisticians usually conduct the statistical analyses, psychological researchers typically conduct their statistical analyses themselves. Although multiple authors on papers have become the *de facto* norm in psychology [24], [25], [26], it is currently unknown how many authors are generally involved in (double-checking) the analyses and reporting of the statistical results. Co-piloting in statistical analysis may concern the independent re-execution of the analyses (e.g., reproducing the results of a test in SPSS), verifying the sample size details, scrutinizing the statistical results in the manuscript, and sharing the data among co-authors before and after publication. In this study, we therefore defined co-piloting as having at least two people involved in conducting the statistical analyses, in writing down the sample details, in reporting the statistical results, and in checking the reported statistical results. In addition, co-piloting in our definition means that at least two people have access to the data before the manuscript is submitted, and that at least two people still have access to the data five years after publication of the article. Data sharing between at least two authors ensures shared responsibility for proper documentation and archiving of the data.

In the present study we estimated the prevalence of inconsistent *p*-values resulting from *t, F, χ^2^, r, Z* and *Wald* tests in articles published in six flagship journals in psychology. To this end, we employed an automated procedure to document the prevalence of statistical reporting errors in 697 articles published in high-impact journals representing six main empirical psychology disciplines. Moreover, we documented the extent to which co-piloting currently occurs in psychology by asking the authors of the articles in our sample a number of questions about the first (or only) study reported in the article: we asked them to indicate whether they had collaborated on four aspects of analyzing data and reporting results, and whether the described data had been shared between the authors. Our design enabled us to fulfill a third objective: to examine the relationship between statistical reporting errors and co-piloting. As we are not aware of any other work documenting collaboration practices on statistical analyses in psychology or any other research area, we had no hypotheses regarding the extent to which co-authors currently employ the co-pilot model. We did however hypothesize that co-piloting is associated with a reduced risk of statistical reporting errors, and thus expected the probability of a given p-value being incorrect to be lower in papers in which the statistical analyses and the reporting of the results had been co-piloted (i.e. where more than one person had been involved). This time-stamped hypothesis can be found at the Open Science Framework via http://osf.io/dkn8a.

## Methods

### The Prevalence of Statistical Reporting Errors

#### Sample

For each psychology subfield as listed in the search engine of Thompson Reuters’ 2012 Journal Citation Reports (Applied Psychology, Biological Psychology, Clinical Psychology, Developmental Psychology, Educational Psychology, Experimental Psychology, Mathematical Psychology Multidisciplinary Psychology, Psychoanalysis, and Social Psychology), we chose the journal with the highest 5-year Impact Factor, which (1) was published in English, (2) required the publication style of the American Psychological Association (APA) [27], and (3) published at least 80 empirical articles in 2011. Four subfields were excluded for different reasons. Educational Psychology was excluded because high-ranking journals in Educational Psychology and Developmental Psychology largely overlapped. Mathematical Psychology was excluded because articles in this field do not usually report the results of NHST. We excluded Multidisciplinary Psychology because we did not regard this field useful to compare subfields of psychology, and we excluded Psychoanalysis because hardly any empirical studies are reported in this field. From the remaining six subfields, the following journals were included in our sample: the *Journal of Applied Psychology* (Applied Psychology), the *Journal of Consulting and Clinical Psychology* (Clinical Psychology), the *Journal of Child Psychology and Psychiatry* (Developmental Psychology), the *Journal of Cognitive Neuroscience* (Experimental Psychology), the *Journal of Personality and Social Psychology* (Social Psychology), and Psychophysiology (Biological Psychology). On 24 October 2012 we downloaded all 775 articles published in these journals since Jan 1^st^, 2012 and then read each abstract in order to determine whether an article was empirical or not. After this selection, our final sample consisted of 697 empirical articles (see Table 1).

**Table 1:** Sample.

Field	Journal title	5-year IF	Articles	Empirical
Applied Psychology	Journal of Applied Psychology (JAP)	6.850	97	78
Biological Psychology	Psychophysiology (PP)	4.049	129	127
Clinical Psychology	Journal of Consulting and Clinical Psychology (JCCP)	6.369	120	105
Developmental Psychology	Journal of Child Psychology and Psychiatry (JCPP)	6.104	114	90
Experimental Psychology	Journal of Cognitive Neuroscience (JCN)	6.268	150	147
Social Psychology	Journal of Personality and Social Psychology (JPSP)	6.901	165	150
Total			775	697

*Note.* 5-yr IF = five-year Impact Factor in 2011. Articles = number of articles published in 2012. Empirical = number of empirical articles published in 2012.

#### Procedure

To assess the accuracy of the *p*-values reported in our sample of articles, we used a recently developed automated procedure called *statcheck* [16], [28]. Statcheck is a package in R, a free software environment for statistical computing and graphics [29], and is available through https://github.com/MicheleNuijten/statcheck. The version of statcheck that we used for this paper (0.1.0) extracts *t, F, χ^2^, r, Z* and *Wald* statistics from articles that are reported as prescribed by the APA Publication Manual [27]. Statcheck re-computes *p*-values in the following way: first, it converts a PDF or HTML file to plain text, and then scans the text for statistical results. Next, it re-computes *p*-values using the test statistics and the degrees of freedom. Finally, it compares the reported and recomputed *p*-value and indicates whether these are consistent or not, while taking into account the effects of rounding. In addition, it specifies whether an inconsistent *p*-value comprises a ‘gross error’: when the *p*-value is inconsistent to the extent that it may have affected a decision about statistical significance (in this case: when it is reported as smaller than 0.05 while the recomputed *p*-value is larger than 0.05, or vice versa). It is important to note that statcheck’s error prevalence estimate may somewhat underestimate or overestimate the true error prevalence because it cannot read statistical results that are inconsistent with the APA’s reporting guidelines [27] or statistical results that contain additional symbols representing for example effect sizes.

In total, 8,110 statistical results were retrieved from 430 of the 697 empirical articles (see Table 2). Five *p*-values that were seemingly reported as larger than 1 were excluded after determining that these had been incorrectly retrieved due to the program’s inability to read *p*-values reported as ‘*p* times 10 to the power of’. A close inspection of the retrieved results revealed that statcheck also had difficulties reading results containing the χ^2^ symbol and results in which effect sizes or other measures had been included between the p-values and the test statistics (e.g. *F*(1, 46) = 8.41, η_p_^2^ = .16, *p* = .006). This explains at least partly why results were retrieved from a relatively low number of articles. For each of the remaining 8,105 retrieved results, two independent coders tracked down whether the test was reported as one-sided or two-sided, and whether the results belonged to the first (or only) study reported in the article or not. Moreover, the two coders manually checked all statistical results that statcheck identified as ‘gross errors’ using a strict coding protocol that required the coders to verify whether these *p*-values indeed constituted an error related to statistical significance. Inter-rater reliability was high: in most cases, both coders agreed on whether the study belonged to Study 1 (Cohen’s Kappa = 0.92) and on whether the results were reported as one-sided or as two-sided (Cohen’s Kappa = 0.85). The inter-rater reliability for decision errors was somewhat lower (Cohen’s Kappa = 0.77), because of possible disagreement on whether the result was tested as one-sided or as two-sided due to ambiguous reporting. Such ambiguity in reporting sidedness of the test highlights the importance of reporting standards, hence we suggest that one-sided tests always be described as “one-tailed”, “one-sided”, or “directional”. In those cases the two coders disagreed on the test’s sidedness, a third coder was asked to independently code the final result.

**Table 2:** Number of articles from which *p*-values were retrieved, number of *p*-values retrieved per journal, and mean number of *p*-values retrieved per article and per journal.

Journal	Nr of articles	Nr of *p*-values retrieved	Mean nr of *p*-values retrieved per article
JAP	42	340	8.10
JCCP	67	833	12.43
JCN	107	1721	16.08
JCPP	39	444	11.38
JPSP	133	4018	30.21
PP	42	749	17.83
Total	430	8105	18.86

*Note*. JAP = Journal of Applied Psychology; JCCP = Journal of Consulting and Clinical Psychology; JCN = Journal of Cognitive Neuroscience; JCPP = Journal of Child Psychology and Psychiatry; JPSP = Journal of Personality and Social Psychology; PP = Psychophysiology.

In the second phase of the protocol, we manually checked the statistical results for which the *p*-value had been reported as ‘*p* = 0.05′. We realized that this was necessary because statcheck could not determine whether a result that had been reported this way was classified as significant by the authors of the article. We therefore looked up all 105 *p*-values reported as ‘ = 0.05′ in the text of the article, determined whether the result had been described as significant or not, and copied the sentence in which the result was reported in into our data file. Again, in those cases where the two coders disagreed (in 2 of the 105 cases), a third coder was asked to independently code the result. For a detailed description of the coding protocol and the flowchart we used, please refer to S1 Table, S1 Figure, and S2 Figure. All manual checks were conducted before the link was made with the survey responses in order to keep the coders blind to whether or not particular analyses were co-piloted.

### Co-Piloting in Psychology

#### Participants

We searched for the contact details of all 3,087 authors of the 697 empirical articles in our sample and obtained at least one email address for each article in our sample. In total, we managed to track down the email addresses of 2,727 authors (88.3%) and sent them an invitation to participate in our online survey in the first week of July, 2013. We sent two reminders to non-responding authors and stopped collecting data one week after sending the second reminder. This way, we aimed to obtain at least one response for most articles. In total, we received at least one response for 346 articles, amounting to an article response rate of 49.6%. Using personalized hyperlinks to the survey (containing the article title and the ‘author number’ indicating whether the respondent was first author, second author, etc.) we were able to establish whether more than one author of an article had responded. To make sure that no more than one response per article was used in the analyses that included survey responses, we only retained the response of the ‘first responding author’, i.e., the author with the lowest author number.

#### Procedure

The online survey was generated using Qualtrics software version 500235 [30]. We programmed the survey in such a way that each respondent was asked the same questions, but that the questions pertained to a specific article published by the individual respondent. In the invitation to the survey, we explicated ethical issues (see below) and stated that survey responses would be linked to the accuracy of the *p*-values in the article with which the survey questions were concerned. In addition, we provided the first author’s email address for respondents to write to if they had further questions before deciding whether to participate.

At the beginning of the survey we encouraged respondents to have the articles near at hand by asking them to indicate how many authors were listed in the paper. Many articles reported more than one study. As different people may have contributed to different studies, the questions would have been difficult or even impossible to answer if they had pertained to all studies. Therefore, the respondents were presented with a set of six questions about the *first or only study* reported in the article asking them to specify who, as indicated by the author number (or ‘other’ category) were involved in: (1) conducting the statistical analyses, (2) writing down the sample details, (3) reporting the statistical results, and (4) checking the reported statistical results. The last two questions in this set pertained to data sharing and asked how many people (5) had access to the data when the manuscript was submitted, and (6) currently have access to the data. These six questions allowed us to construct six corresponding ‘co-piloting’ variables: if only one person was involved, the variable was coded ‘0’ (not co-piloted), if two or more persons were involved, the variable was coded ‘1’ (co-piloted). Finally, we asked respondents whether they wished to receive a report about the accuracy of the *p*-values reported in their article, and whether they wished to participate in a raffle in which they could win one of five $100 Amazon.com vouchers. The invitation e-mail and the survey itself can be found at the Open Science Framework via http://osf.io/ncvxg.

### The Relationship between Co-Piloting and Statistical Reporting Errors

**Figure 1:**
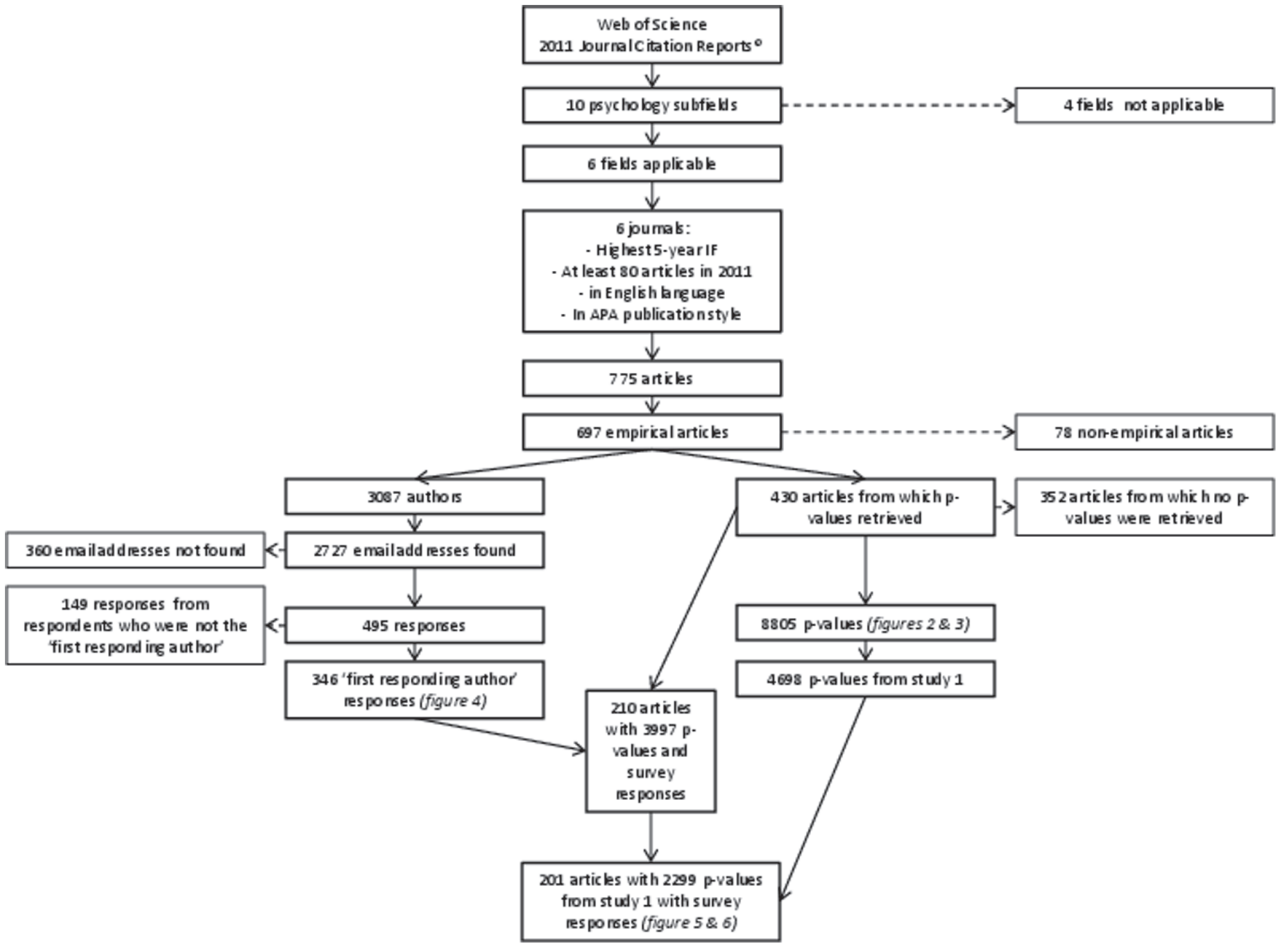
Flow chart for composition of sample.

To analyze the relationship between co-piloting and the accuracy of *p*-values reported in the first or only study in the corresponding articles, we merged the data file containing the retrieved *p*-values from each article and the data file containing the survey responses. While *p*-values had been retrieved from 430 out of 697 articles, and survey responses were obtained for 346 articles, these sets of articles did not completely overlap (i.e., for some articles statistical results were retrieved but no survey response was obtained, and vice versa). In total, the data of 210 articles (48.8% of the 430 articles from which statistical results had been retrieved) could be matched. Thus, we matched each statistical result retrieved from the first (or only) study reported in these articles to the survey responses given by the respondent with the lowest author number. The statistical results of the remaining 220 articles were retained in the file to analyze the effect of non-response. A schematic overview of our sample is presented in Fig. 1. Based on the study of Wicherts, Bakker, and Molenaar [14] who found a relationship between willingness to share research data and the prevalence of reporting errors in a sample of 48 articles, we expected to have enough power to detect a relationship between co-piloting and statistical reporting errors in our sample of 430 papers from which p-values were retrieved. With the 210 articles for which we obtained survey responses and had retrieved p-values, our sample was still more than four times as large as in Wicherts et al.’s study [14].

### Ethics Statement

This study was approved by the ethics committee of the Tilburg School of Social and Behavioral Sciences under the following conditions: (1) specific errors uncovered during this study would not be discussed in publications, presentations, writing or conversation with others, (2) the survey responses would be processed anonymously, and (3) survey respondents would receive feedback about the accuracy of the *p*-values in their article if they wished. In total, 384 respondents requested and received feedback via email. Respondents provided informed consent by ticking ‘yes’ at the statement ‘I have read and understood the above and agree to participate’ on the introductory page of the survey.

### Statistical Analysis

We uploaded our analytical plan regarding the confirmatory analyses at the Open Science Framework before the survey data collection started, which can be viewed via https://osf.io/qutsy. As our main hypotheses were tested by six analyses each, we corrected our alpha levels in these analyses for multiple testing by dividing.05 by six. All analyses were conducted by at least two of the authors to reduce the probability of any errors on our own part. All scripts used to prepare the data files, to conduct the analyses and to construct the graphs, to anonymize our data, and to draw the winners of the raffle can be found on the Open Science Framework via http://osf.io/ekush.

### Data Availability

The first, non-merged anonymous survey data file can be viewed via http://osf.io/4bvqh. The data on the Open Science Framework are open access with no copy right issues and can be accessed by readers in the same manner as the authors. The second, merged data file contains *p*-values that can be traced back to individual articles, and can therefore not be shared without restrictions imposed by our ethics committee. The Psychology Ethics Committee of the Tilburg School of Social and Behavioral Sciences approved this study under the strict condition that we would not make these data file publicly available. We will however share these data after written agreement, and only with other researchers wishing to verify our results (see Article 8.14 of the APA ethical principles of psychologists and code of conduct [31]). Requests for data can be sent to the authors Coosje L. S. Veldkamp (C.L.S.Veldkamp@tilburguniversity.edu) or Jelte M. Wicherts (J.M.Wicherts@tilburguniversity.edu).

## Results

### The Prevalence of Statistical Reporting Errors

Our first aim was to estimate the prevalence of statistical reporting errors in journals representing different areas of psychology using an automated procedure. First, we present the error rates at the article level: what is the probability that an article contains at least one *p*-value that comprises an error? As the dependent variable was dichotomous (the article does or does not contain at least one inconsistent *p*-value), we carried out simple logistic regression analyses (intercept only models) to estimate the probabilities and their 95% confidence intervals (CI). The results collapsed over all journals revealed that almost two out of three articles (63.0%, CI [58.4–67.5]) contained at least one *p*-value that comprised an error, and that one in five articles (20.5%, CI [16.9–24.5] contained at least one *p*-value that comprised a gross error.

**Figure 2:**
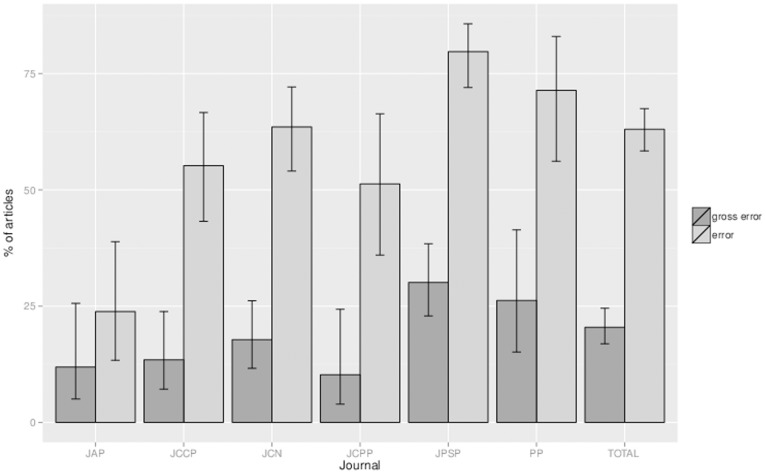
The probability per journal that an article contains at least one *p*-value comprising an error or gross error (with 95% confidence interval). *Note.* JAP = Journal of Applied Psychology (*n* = 42); JCCP = Journal of Consulting and Clinical Psychology (*n* = 67); JCN = Journal of Cognitive Neuroscience (*n* = 107); JCPP = Journal of Child Psychology and Psychiatry (*n* = 39); JPSP = Journal of Personality and Social Psychology (*n* = 133); PP = Psychophysiology (*n* = 42); TOTAL = all articles together (N = 430).

We also compared the error prevalence across different journals/fields. Logistic regression analyses with journal as predictor revealed that there were differences in error rates between the journals: χ*^2^* (5, *N* = 430) = 49.46, *p*<.001. The probability that an article contained at least one *p*-value that comprised an error was lower in the *Journal of Applied Psychology* than in all other journals (23.8%, CI [13.3−38.9], all *p*s ≤.002<0.05/6) except the *Journal of Child Psychology and Psychiatry* (51.3%, CI [36.0–66.4], *p* = .012>0.05/6). At the same time, this probability was higher in *the Journal of Personality and Social Psychology* (79.7%, CI [72.0−85.7], all *p*s ≤.006<0.05/6) than in all other journals except *Psychophysiology* (71.4%, CI [56.1–83.0], *p* = .264>0.05/6). These differences may be attributable to differences between journals in the mean number of reported *p*-values per article, as a higher number of reported *p*-values entails a higher probability that an article contains an error. For example, an article in the *Journal of Personality and Social Psychology* contains more than 30 *p*-values on average, whereas the average article in the *Journal of Applied Psychology* contains only slightly more than eight *p*-values. The probability that an article contained at least one *p*-value that comprised a gross error differed also by journal: χ*^2^* (5, *N* = 430) = 15.46, *p* = .009, but no journal differed significantly from any other journal (all *p*s ≥0.012>0.05/6). The error probabilities for the sample as a whole and for each field separately are presented in Fig. 2, together with their 95% confidence intervals.

Next, we present the results at the level of the individual *p*-value: i.e. what is the probability that a given *p*-value comprises an error? Because the dependent variable was again dichotomous (the *p*-value is either inconsistent or not) and because the *p*-values are nested within their articles, we carried out multilevel logistic regression analyses with article as random factor to estimate the probabilities and their 95% confidence intervals (CI). The results collapsed over all *p*-values showed that approximately one in ten *p*-values comprised an error (10.6%, CI [9.4–11.9]) and one in 125 *p*-values comprised a gross error (0.8%, CI [0.6–1.0]).

**Figure 3:**
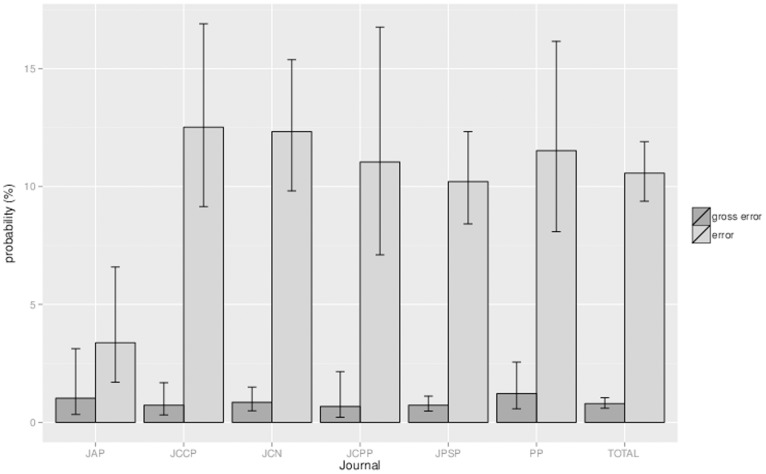
The probability per journal that a given *p*-value comprises an error or a gross error (with 95% confidence interval). *Note*. JAP = Journal of Applied Psychology (*n* = 340); JCCP = Journal of Consulting and Clinical Psychology (*n* = 833); JCN = Journal of Cognitive Neuroscience (*n* = 1,721); JCPP = Journal of Child Psychology and Psychiatry (*n* = 444); JPSP = Journal of Personality and Social Psychology (*n* = 4,018); PP = Psychophysiology (*n* = 749); TOTAL = all *p*-values together (N = 8,105).

Running the multilevel logistic regression analyses with article as a random factor and journal as a fixed factor revealed that there were differences in the *p*-values’ error probabilities between journals: χ*^2^* (5, *N* = 8105) = 17.53, *p* = .004. The probability that a given *p*-value comprised an error was lower in the *Journal of Applied Psychology* (3.4%, CI [1.7–6.6]) than in all other fields (all *p*s ≤.004<0.05/6). One explanation for the lower error probability in this journal may be that its low mean number of reported *p*-values per article (8.10) renders errors more easily detectable by (co-)authors and other readers. The probability that a *p*-value comprised a gross error did not differ between journals: χ*^2^* (5, *N* = 8105) = 1.92, *p* = .860. The error probabilities for the sample of *p*-values as a whole as well as for the *p*-values in each field separately are presented in Fig. 3, together with their 95% confidence intervals.

One may notice that for the *Journal of Applied Psychology (JAP)* the probability that a *p*-value comprises a gross error seems relatively high compared to the overall probability that a *p*-value in *JAP* comprises an error. However, we tested if the conditional probability of a gross error given an error was different across journals, and this was not the case: χ*^2^*(5, *N* = 1149) = 9.09, *p* = .106.

### Co-Piloting in Psychology

**Figure 4:**
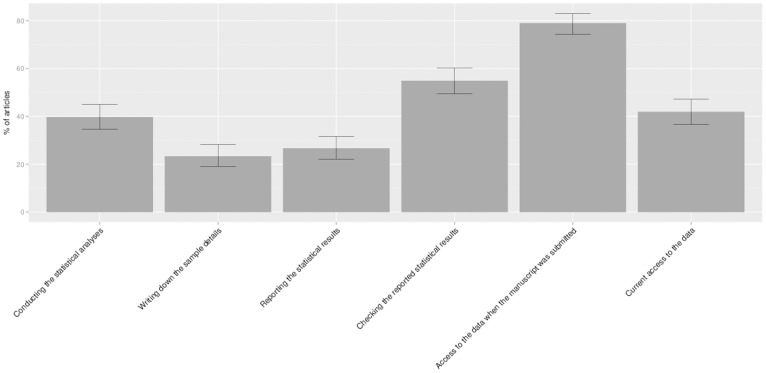
The percentage of articles in which co-piloting occurred for various processes (with 95% confidence intervals). *Note*. statistical analyses = conducting the statistical analyses (N = 335); write up sample details = writing the sample details in the manuscript (N = 330); write up results = writing up the results in the manuscript (N = 334); check results in manuscript = checking of the results in the manuscript by someone other than the person who wrote up the results in the manuscript (N = 326); data sharing at submission = having access to the data at the moment the manuscript was submitted (N = 333); data sharing now = having access to the data at the moment the survey was being filled in (N = 332).

Our second aim was to document the extent to which co-piloting currently occurs in psychological research. To answer this, we computed a co-piloting variable for each of the six co-piloting questions in the survey. Specifically, we computed for each of the processes (analyzing the data, writing down the sample details in the manuscript, writing down the statistical results in the manuscript, checking the results written down in the manuscript, sharing the data among co-authors before submission, and archiving the data after submission) how many people had been involved and coded those parts in which two or more people had been involved as 1 (co-piloted), and those parts in which only one person had been involved as 0 (not co-piloted). We ran a logistic regression analysis (intercept model only) to estimate the probabilities and 95% confidence intervals for each of the six processes (see Fig. 4). Because journal was no significant predictor in any of the six analyses (all *p*s ≥.015>0.05/6), the results presented in Fig. 4 are collapsed over journals. Note that the sample sizes for the individual analyses differed slightly due to some missing values that were excluded pairwise (see note below Fig. 4).

As can be seen in Fig. 4, the statistical analyses were most often conducted by one person only: co-piloting occurred in just 39.7% of the articles (CI [34.6%–45.0%]. Similarly, in most articles, only one person wrote down the sample details and the statistical results in the manuscript (co-piloting occurred in 23.3% (CI [19.1%−28.2%]) and 26.6% (CI [22.2%−31.6%]) of the articles, respectively). However, the results of the analyses as written down in the manuscript were checked by a second person slightly more often than not (54.9%, CI [49.5%−60.2%). On the other hand, in most articles, the data had been shared with at least one other person when the manuscript was submitted (79.0%, CI [74.3%−83.0%], meaning that at least one other person had the opportunity to look at the data set before the article was published. Less than two years after publication however, data storage by more than one person occurred only in the minority of cases (41.9%, CI [36.7%−47.2%].

### The Relationship between Co-Piloting and Reporting Errors

**Figure 5:**
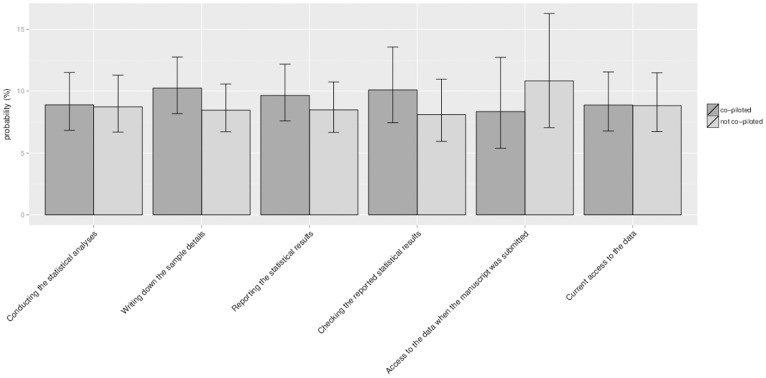
The probability that a *p*-value in the first (or only) study reported comprises an error: co-piloted studies versus non-co-piloted studies. *Note*. statistical analyses = conducting the statistical analyses (N = 2,247); write up sample details = writing the sample details in the manuscript (N = 2,215); write up results = writing up the results in the manuscript (N = 2,231); check results in manuscript = checking of the results in the manuscript by someone other than the person who wrote up the results in the manuscript (N = 2,185); data sharing at submission = having access to the data at the moment the manuscript was submitted (N = 2,228); data sharing now = having access to the data at the moment the survey was being filled in (N = 2,226).

**Figure 6:**
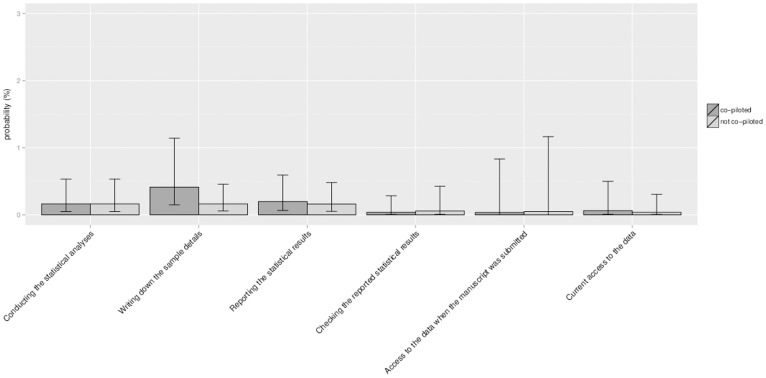
The probability that a *p*-value in the first (or only) study reported comprises a gross error: co-piloted studies versus non-co-piloted studies. *Note*. statistical analyses = conducting the statistical analyses (N = 2,247); write up sample details = writing the sample details in the manuscript (N = 2,215); write up results = writing up the results in the manuscript (N = 2,231); check results in manuscript = checking of the results in the manuscript by someone other than the person who wrote up the results in the manuscript (N = 2,185); data sharing at submission = having access to the data at the moment the manuscript was submitted (N = 2,228); data sharing now = having access to the data at the moment the survey was being filled in (N = 2,226).

Our third aim was to establish whether a relationship exists between co-piloting and the probability that a *p*-value comprised an error. To answer this question, we only took into account those *p*-values of articles of which at least one author responded to our survey. By means of a multilevel logistic regression analysis with article as random factor and journal as fixed factor we first established that in this subsample there were no differences between journals in the probability that a *p*-value comprised an error (χ*^2^* (5, *N* = 2299) = 6.35, *p* = .274), nor in the probability that a *p*-value comprised a gross error (χ*^2^* (5, *N* = 2299) = 4.15, *p* = .528). We then ran six different multilevel logistic regression analyses, each with article as random factor, and one of the six co-piloting variables as fixed factor. Our hypothesis that co-piloting is related to the probability that a given *p*-value was associated with a reduced error risk lacked support: we found no differences for any of the six processes between articles in which co-piloting had occurred and articles in which co-piloting had not occurred in the probability that a given *p*-value was inconsistent (all *p*s ≥.283>0.05/6, see Fig. 5), nor in the probability that a *p*-value was inconsistent to the extent that it may have affected a decision about statistical significance (all *p*s ≥.323>0.05/6, see Fig. 6).

### Non-Response

Finally, we studied the effect of non-response by comparing the error probabilities in articles for which we obtained survey responses to the error probabilities in articles for which we did not obtain survey responses. Responses to the survey were not significantly associated either with the probability that an article contained at least one *p*-value that comprised an error (Wald *Z* = -0.882, *p* = .378), or with the probability that an article contained at least one *p*-value that comprised a gross error (Wald *Z* = -1.308, *p* = .191). These results indicate that the probability that an article contained at least one *p*-value that comprised an error did not seem to be associated with whether the authors responded to the survey.

At the *p*-value level, there was an effect of response on the probability that a given *p*-value comprised an error (Wald *Z* = -2.194, p = .028), but there was no effect of response on the probability that a given *p*-value comprised a gross error (Wald *Z* = -1.819, *p* = .069). In other words, these results indicate that the probability that a *p*-value comprised an error was higher in articles published by authors who did not respond to the survey than in articles published by authors who did respond to the survey, but that no such association was found with respect to the probability that a *p*-value comprised a gross error.

## Discussion

We estimated the prevalence of inconsistent *p*-values in six top psychology journals by means of an automated procedure to retrieve and check errors in the reporting of statistical results, in order to replicate earlier estimates of error rates in the psychological literature [11], [15], [16], [17]. Our results show a somewhat higher probability for articles to contain at least one *p*-value that comprises an error compared to the two studies by Bakker and Wicherts (63% vs. 45% [16] and 50% [11]), and a higher probability for articles to contain at least one *p*-value that comprises a gross error (20% vs. 15% [11], [16]). Our error probability estimates at the article level may be somewhat higher because the top journals in our sample typically require more than one study and hence include the results of more tests than the lower-ranked journals in Bakker and Wicherts’ [11] study. Our estimate of the probability that a *p*-value comprises an error was in between the estimates of Bakker and Wicherts: 10% vs 7% [16] and 18% [11]). A possible explanation for the difference with the higher estimate of 18% [11] is that in the first study by Bakker and Wicherts [11], statistical results that were not exactly reported as prescribed by the APA manual [27] were counted as errors, whereas in their later study [16] and in our study, this type of error was not taken into account. On the other hand our error prevalence estimates may have been somewhat inflated by excluding reported statistical results that included an effect size. If we would assume that reporting effect sizes is associated with more knowledge about statistics, authors who reported effect sizes may have made fewer mistakes in reporting. In any case, our estimates are alarmingly high: almost two out of three of the articles published in one of these flagship journals contain at least one statistical reporting error and one in every ten reported p-values is inconsistent with the reported test statistic and the accompanying degrees of freedom.

Moreover, we documented the extent to which co-piloting currently occurs in psychology. Ours is the first study that looked at how often psychology researchers work together on the analyses and reporting of results and at how often data are shared among co-authors. Although 99.1% of the articles had more than one author, not all co-authors appear to feel shared responsibility for the accuracy of the data analysis. In most articles the analyses were conducted by one person only and the results in the manuscript were checked only slightly more often than not by a co-author or someone else. We realize however that ‘checking the results in the manuscript’ may not actually constitute re-analysis or recalculation of the *p*-values and that the term ‘checking the results’ may therefore have been somewhat ambiguous. Yet data sharing among co-authors seems quite common: the results indicated that data from four out of five articles had been shared among at least two authors at the time the manuscript was submitted. This means that at least one co-author had the opportunity to have a look at the data file before submission, although this does not mean that they have actually done so, and if they did, in what way they inspected the data. On the other hand, we find it rather disconcerting that even if the data were shared before submission, the data of more than half of the published articles are currently stored by one person only. If the data are stored in a safe place (e.g., in a data repository, or in the ‘cloud’), this may not constitute an archiving problem (specifically if they are well documented). However, if the data are stored on one researcher’s hard drive, the risk of loss of the data is considerable. Recent results show that the availability of research data declines rapidly over time [32], notwithstanding that ethical guidelines [27] and professional standards require the archiving of data for at least five years after publication. Sharing data with co-authors requires rigorous documentation, which is likely to increase the chances that data are still available for re-analyses, verification, and further use in the future [33], [34], [35], [36]. Finally, our survey results show that in the majority of articles only one author wrote down the sample details and the statistical results. However, one could argue that these two variables may not have captured the concept of co-piloting very well, as it may not have been clear to our respondents how we actually envisioned co-piloting on actual writing. Our intention was to measure whether the sample size details and the results were discussed between authors before/during the actual writing process, as we believe that some errors may occur in this phase and may be reduced by discussion of the results and the output of the analyses among co-authors. Such aspects should be subject to further study. Another interesting avenue for further research would be a more fine-grained analysis of specific roles of each author and potential differences between responding authors in their responses to co-piloting questions.

Finally, we looked at whether co-piloting on statistical analyses, reporting of results, and data sharing among co-authors was associated with a reduced risk of statistical reporting errors. Contrary to our expectations, we did not find support for this relationship. A relationship may simply not exist, but we believe that the relationship may have been obscured by confounding mechanisms. For instance, our reliance on self-report may have produced desirable responses (in this case, answers indicating shared responsibility). The fact that we asked respondents to indicate *which authors* were involved in each part of the processes rather than asking *how many* people were involved may have also rendered the survey more sensitive. Another factor that may have played a role is that the difficulty of the statistical analyses may have increased both the error probability of the reported statistical result and the probability that the authors collaborated on the statistical analyses, which may have offset the effect of collaboration on the error probability. Finally, the finding that the probability that a given *p*-value was inconsistent was higher in articles of which the authors did not respond to our survey may be an indication that authors who worried that some of their *p*-values might turn out inconsistent were less inclined to respond to our survey. Note, however, that the relation between responses and inconsistent *p*-value probability was weak. Finally, because we could only use those statistical results that were part of the first or only study reported in the articles and because those results could not always be matched to survey responses, our sample size (and hence our statistical power) turned out lower than we expected.

Even if co-piloting turns out not to be associated with a reduced risk of statistical reporting errors, we do believe that co-piloting helps to intercept other human errors in the use of statistics and in scientific research in general. The risk of many forms of slips and lapses, to which experts in any field are particularly prone [19] should diminish considerably when more than one person is involved [20], [21]. In addition, co-piloting may benefit science by requiring transparency: co-piloting among co-authors requires proper data documentation, data archiving, openness, and discourse about statistical and methodological decisions. Most articles concerning data sharing focus on data sharing with people outside the research group [33], [35], [37], [38], [39], but we believe that sharing of data as well as of methodological and statistical decisions ought to start within the research group, i.e., among co-authors themselves. Even if full co-piloting as defined in this article is not feasible due to time or other constraints, we encourage authors to implement at least some double-checking of data files, analysis scripts, and results into their routines. For example, this double-checking could be part of regular PhD-student supervision, fostering acuity on the part of both the student and the supervisor(s). At the same time, such a practice would set an example in emphasizing the importance of meticulousness in data analysis and reporting.

There have been suggestions for journal editors to increase author accountability by requesting a description of author contributions to each stage of the research process [40], a policy that enhances transparency and accountability and has now been adopted by a number of journals including the *Journal of the American Medical Association, The Lancet, PLoS ONE,* and *Psychological Science*. Finally, reporting confidence intervals and effect sizes as suggested by many statisticians trying to improve the use and reporting of statistics (e.g. [7]) and as prescribed by the APA’s updated reporting guidelines [27] may reduce error rates as this requires more scrutiny in interpreting results and may allow (co-)authors (and other readers) to quickly spot striking inconsistencies between reported numbers.

Like all fields of science, psychological science depends on the accuracy of the results reported in its literature. Human error of all forms is a part of science, but scientists nonetheless have the responsibility to eliminate as much error as possible.

## Supporting Information

S1 Figure Manual check of decision errors part 1. *Note*. S_1_ = study 1, E_1_ = one-sided error, E_2_ = two-sided error, D_1_ = one-tailed decision error, D_2_ = two-tailed decision error, P_rep_ = reported *p*-value, P_real_ = computed/real *p*-value.
(TIFF)

S2 Figure Manual check of decision errors part 2. Note. S1 = study 1, E1 = one-sided error, E2 = two-sided error, Prep = reported p-value, Pcomp = computed/real p-value.
(TIFF)

S1 Table Coding protocol.
(DOCX)
