# Supplementary figures and images for: Statistical Reporting Errors and Collaboration on Statistical Analyses in Psychological Science
Source: PLoS One. 2014 Dec 10;9(12):e114876. doi: 10.1371/journal.pone.0114876 (PMC4262438; doi:10.1371/journal.pone.0114876)

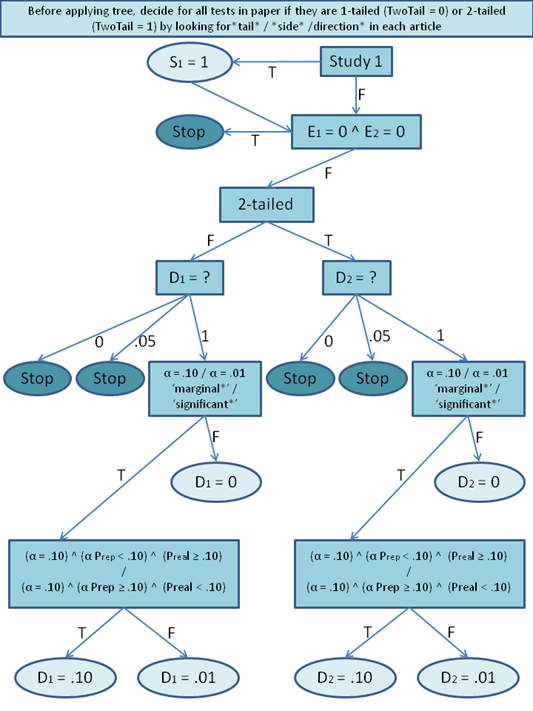

Supplement: S1 Figure — Manual check of decision errors part 1. Note. S1 = study 1, E1 = one-sided error, E2 = two-sided error, D1 = one-tailed decision error, D2 = two-tailed decision error, Prep = reported p-value, Preal = computed/real p-value. (TIFF) [file pone.0114876.s001.tiff]

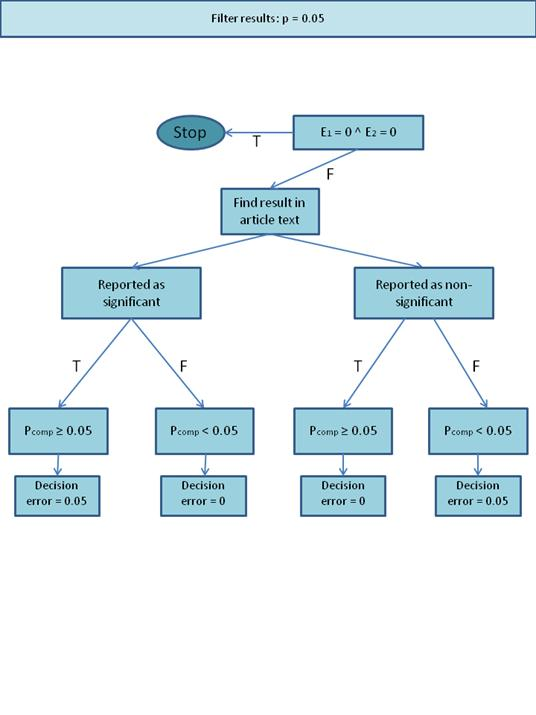

Supplement: S2 Figure — Manual check of decision errors part 2. Note. S1 = study 1, E1 = one-sided error, E2 = two-sided error, Prep = reported p-value, Pcomp = computed/real p-value. (TIFF) [file pone.0114876.s002.tiff]
