# Supplementary material for: Statistical Reporting Errors and Collaboration on Statistical Analyses in Psychological Science
Source: PLoS One. 2014 Dec 10;9(12):e114876. doi: 10.1371/journal.pone.0114876 (PMC4262438; doi:10.1371/journal.pone.0114876)
Supplement: S1 Table — Coding protocol. (DOCX) [file pone.0114876.s003.docx]

Table S1.

*Coding protocol*

| **Variable** | **How entered** | **Coding** | **Explanation** |
| --- | --- | --- | --- |
| Source | statcheck | String | Filename as coded by student assistant. This includes journal, author, year, volume, issue, page number of first page, and title. |
| Statistic | statcheck | t, F, r, X2, Z, Wald | Test 'statistic'. In this case, r and Wald are also counted as test statistics. |
| df1 | statcheck | Numerical | Reported degrees of freedom. |
| df2 | statcheck | Numerical | Reported degrees of freedom. |
| Test.Comparison | statcheck | <, >, = | How is the test statistic reported? E.g. t(10) < 1, t(10) > 1 or t(10) = 1. |
| Value | statcheck | Numerical | Value of the test statistic. |
| Reported.Comparison | statcheck | <, >, =, ns | How is the p value reported? P < .05, p > .05, p = .05, or ns for not significant. |
| Reported.P.Value | statcheck | Numerical | Reported *p*-value. |
| Computed | statcheck | Numerical | Computed *p*-value. |
| Raw | statcheck | String | The raw result as read by statcheck. |
| Error | statcheck | Logical (1, 0) | 1 if the reported p value is incongruent with the computed p value (else 0). |
| DecisionError | statcheck/manually | 0, 0.01, 0.05,0.1, 1 | A decision error occurs when a reported significant result is not significant after recomputation or when a reported non-significant result is actually significant Statcheck: 0=no decision error, .05= decision error at alpha is .05, 1=decision error at alpha is .10 or .01 Manually: when DecisionError=1, check the actual level of significance in paper. If it's not mentioned, fill in 0. If it is, fill in the reported level of significance. |
| CopyPaste | statcheck | Logical (1, 0) | 1 if the exact string of the extracted raw results also occurs somewhere else in the article. |
| Error_OneTail | statcheck | Logical (1, 0) | 1 if the reported p value is incongruent with the one sided computed p value (else 0). |
| DecisionError_OneTail | statcheck/manually | 0, 0.01, 0.05,0.1, 1 | A decision error occurs when a reported significant result is not significant after recomputation or when a reported non-significant result is actually significant Statcheck: 0=no one tailed decision error, .05= one tailed decision error at alpha is .05, 1= one tailed decision error at alpha is .10 or .01 Manually: when DecisionError_OneTail=1, check the actual level of significance in paper. If it's not mentioned, fill in 0. If it is, fill in the reported level of significance. |
| TwoTailed | statcheck/manually | Logical (1, 0) | 1 if the test is two tailed (default). Manually change it to 0 if the test is one tailed. |
| Study 1 | Manually | Logical (1, 0) | 1 if the result belongs to study 1 (0=default). Manually change it to 1 if the result belongs to study 1. |
| Coder 1 | manually | Initials | Who coded the article the first time? Fill in your initials (e.g., MN for Michèle Nuijten) |
| Coder 2 | manually | Initials | Who checked the article the second time? Fill in your initials (e.g., CV for Coosje Veldkamp) |
| Check error coder 1 | manually | Logical (1, 0) | If statcheck reported an error, check this manually. 1 if the result really is an error. 0 if the result is correct and statcheck wrongly classified it as error. |
| Check error coder 2 | manually | Logical (1, 0) | If statcheck reported an error, check this manually. 1 if the result really is an error. 0 if the result is correct and statcheck wrongly classified it as error. |
